# Target Therapy With Vaccinia Virus Harboring IL-24 For Human Breast Cancer

**DOI:** 10.7150/jca.37590

**Published:** 2020-01-01

**Authors:** Lili Deng, Jun Fan, Yuedi Ding, Xue Yang, Biao Huang, Zhigang Hu

**Affiliations:** 1 NHC Key Laboratory of Nuclear Medicine, Jiangsu Key Laboratory of Molecular Nuclear Medicine, Jiangsu Institute of Nuclear Medicine, Wuxi 214063, China.; 2 Wuxi Children's Hospital, Wuxi People's Hospital affiliated to Nanjing Medical University, Wuxi 214023, China.; 3 School of Life Science, Zhejiang Sci-Tech University, Hangzhou 310018, China.

**Keywords:** oncolytic vaccinia virus, interleukin-24, breast cancer, gene therapy, apoptosis.

## Abstract

**Background:** Breast cancer is a heterogeneous disease with high aggression and novel targeted therapeutic strategies are required. Oncolytic vaccinia virus is an attractive candidate for cancer treatment due to its tumor cell-specific replication causing lysis of tumor cells as well as a delivery vector to overexpress therapeutic transgenes. Interleukin-24 (IL-24) is a novel tumor suppressor cytokine that selectively induces apoptosis in a wide variety of tumor types, including breast cancer. In this study, we used vaccinia virus as a delivery vector to express IL-24 gene and antitumor effects were evaluated both *in vitro* and *in vivo*. **Methods:** The vaccinia virus strain Guang9 armed with IL-24 gene (VG9-IL-24) was constructed via disruption of the viral thymidine kinase (TK) gene region. The cytotoxicity of VG9-IL-24 in various breast cancer cell lines was assessed by MTT and cell cycle progression and apoptosis were examined by flow cytometry. *In vivo* antitumor effects were further observed in MDA-MB-231 xenograft mouse model. **Results:*** In vitro,* VG9-IL-24 efficiently infected and selectively killed breast cancer cells with no strong cytotoxicity to normal cells. VG9-IL-24 induced increased number of apoptotic cells and blocked breast cancer cells in the G2/M phase of the cell cycle. Western blotting results indicated that VG9-IL-24-mediated apoptosis was related to PI3K/β-catenin signaling pathway. *In vivo,* VG9-IL-24 delayed tumor growth and improved survival. **Conclusions:** Our findings provided documentation that VG9-IL-24 was targeted* in vitro* and exhibited enhanced antitumor effects, and it may be an innovative therapy for breast cancer.

## Introduction

Breast cancer is the principal cause of cancer-related death in women. Despite the development in early diagnostics and therapies, the overall survival rates remain unchanged during last two decades 1. Specially, triple-negative breast cancer (TNBC), an aggressive subtype of breast cancer with poorest prognosis and lower survival, is refractory to current therapies duo to lack expression of estrogen receptor (ER), progesterone receptor (PR) and the overexpression of the HER2/neu receptor. Traditional therapies for breast cancer, such as surgery, chemotherapy and radiotherapy, have a series of adverse effects including inefficient curative effects, cognitive impairments, tumor metastasis increase and resistance to established therapies. Therefore, novel therapeutic strategies for breast cancer therapy are strongly required.

Oncolytic vaccinia virus represents a potential strategy for cancer therapy due to its appealing features such as extensive safety as a live vaccine and efficient delivery to metastatic tumors 2-4. Besides, oncolytic vaccinia virus can also be used as gene expression carrier due to its large size and expression using its own enzyme systems. With the replication of vaccinia virus, the copies of carried genes are also increased, leading to efficient expression levels in tumor tissues. So far, vaccinia virus has been modified to carry various antigens, cytokines, and immunostimulatory molecules, such as Wyeth strain modified by insertion of granulocyte microphage colony-stimulating (GM-CSF) 5, and Western Reserve strain armed with interleukin (IL)-12^6^, IL-4 7, IL-10 8 and IFN-β 9.

Recently, interleukin family member IL-24 has been attracted researchers attentions by the virtue of the effect on human melanoma cell growth and differentiation 10. Preclinical studies have shown that IL-24 induces apoptosis in various cancer cells but has no significant cytotoxicity to normal cells 11, 12. Traditional delivery of IL-24 by liposome or replication-defective adenovirus cannot target tumor cells, which limits its value on cancer gene therapy. Therefore, in this study, we used vaccinia virus strain Guang9 (VG9) as a delivery vector to express IL-24 gene. The antitumor effects and therapeutic potential of VG9-IL-24 for breast cancer were evaluated both *in vitro* and *in vivo*.

## Materials and Methods

### Cell lines

The human breast cancer cell lines MDA-MB-231 (TNBC), MDA-MB-453 (TNBC), MDA-MB-468 (TNBC), MCF-7 (ER+, PR+), SK-BR-3 (HER2+), the human normal breast epithelial cell line MCF-10A, and the human embryonic kidney cell line HEK-293 were purchased from Shanghai Cell Collection (Shanghai, China). African green monkey kidney epithelial cell lines Vero and BSC-40 were purchased from the American Type Culture Collection (ATCC; Manassas, VA, USA). All cells were cultured under the conditions suggested by ATCC.

### Construction, identification and titration of VG9-IL-24

The recombinant vaccinia virus carrying IL-24 gene was constructed via homologous recombination between shuttle plasmid (pCB, gifted from Professor Liu) and vaccinia virus strain VG9 (obtained from National Institutes for Food and Drug Control, Beijing, China), which was derived from Chinese vaccine strain Tian Tan (VTT) and has been demonstrated be more attenuated 13. The IL-24 gene (full-length cDNA was purchased from Sino Biological Inc. , Beijing, China) was inserted into viral thymidine kinase (TK) locus and was under the control of the vaccinia synthetic early/late promoter 14. Recombinants were selected in Vero cells via xanthine-guanine phosphoribosyltransferase (XGPRT) selection. Viral genomes were extracted by Generay kit (Shanghai Generay Biotech Co., Ltd, Shanghai, China). The presence of the inserted IL-24 gene was verified by polymerase chain reaction (PCR) using IL-24 primers (P1: 5'-AGATCTATGAGTTGGGGAC-3'; P2: 5'- GAATTCTCAGAGATGGTAG-3'). A pure recombinant was also verified by PCR using primers external to the site of recombination (P1: 5'- ATGAACGGCGGACATATTCA-3'; P2: 5'-TTATGAGTCGATGTAACACTTTC-3'). The results were visualized by ethidium bromide agarose gel electrophoresis. Recombinant vaccinia virus stock were amplified in Vero cells and purified over a sucrose gradient centrifugation. Plaque-forming unit (PFU) virus titers (PFU/ml) were determined by plaque assay on BSC-40 cells after three cycles of freezing and thawing. The TK-deleted recombinant vaccinia virus expressing enhanced green fluorescent protein (EGFP) gene was generated as control and the construction was described previously 15.

### IL-24 expression

The human breast cancer cell lines and normal breast cells grown in 12-well plates were infected with 0.1 multiplicity of infection (MOI) of VG9-IL-24 for 24 h. Supernatants and lysates were collected and IL-24 levels were determined by enzyme-linked immunosorbent assay (ELISA; R&D systems Inc., Minneapolis, MN, USA) according to the manufacturer's manual.

### Cytotoxicity assay

The human breast cancer cell lines MDA-MB-231, MDA-MB-453, MDA-MB-468, MCF-7, SK-BR-3, and the human normal breast cell line MCF-10A were seeded in 96-well plates at the density of 1×10^4^/well. After infection with different concentrations of virus for 72 h, cell viability was analyzed by the MTT cytotoxicity assay.

### Detection of apoptotic cells

Hoechst 33258 staining assay was carried out to observe morphological characteristics of apoptotic cells. MDA-MB-231 cells and MCF-10A cells were seeded in 12-well plates. After infection with VG9-IL-24, VG9-EGFP or PBS for 48 h, cells were incubated with Hoechst 33258 (Beyotime Biotechnology, China) for 30 min. The apoptotic morphological changes of cells are observed under Olympus IX51 fluorescence microscope immediately.

The ratios of apoptotic cells were determined by flow cytometric analysis using an Annexin V/propidium iodide (PI) apoptosis detection kit (Roche Applied Science, Germany). MDA-MB-231 cells were harvested after infection with VG9-IL-24, VG9-EGFP or PBS for 48 h. Aliquots of cells were resuspended in 1 ml binding buffer mixed with 20 μl of fluorescein isothiocyanate (FITC)-labeled Annexin V and 20 μl of PI, and put in the dark at room temperature for 10 min. Flow cytometry (BD, FACSCalibur, USA) was performed immediately after staining.

### Cell cycle analysis

MDA-MB-231 cells seeded in 6-well plates were infected with VG9-IL-24, VG9-EGFP or PBS for 48 h, then were harvested and fixed in 70% cold ethanol overnight at -20 ℃. Cells were washed with PBS and resuspended in 50 μg/ml of PI solution. After incubation for 30 min in the dark at 37 ℃, the treated cells were analyzed by flow cytometry (BD, FACSCalibur, USA). The percentages of G0/G1, S, and G2/M stage cells were quantified using Flow Jo Software (Tristar, CA, USA).

### Western blot analysis

Cells infected with virus or mock-infected cells were harvested and lysed with RIPA lysis buffer containing protease and phosphatase inhibitors (Halt Protease Inhibitor Cocktail, Thermo Fisher Scientific Inc.). Equal amounts of protein from each sample were subjected to electrophoresis on SDS-polyacrylamide gel and transferred to polyvinylide difluoride membrane (Thermo Scientific™, USA). Rabbit monoclonal antibody for PI3K (Cell Signaling Technology, Danvers, MA, USA), β-catenin (Beyotime Institute of Biotechnology, China), phospho-Akt (Ser^473^), Akt (Cell Signaling Technology, Danvers, MA, USA), phospho-GSK-3β (Ser^9^), GSK-3β (Beyotime Institute of Biotechnology, China), Bxl-xL (Beyotime Institute of Biotechnology, China) and mouse monoclonal antibodies for β-actin (Santa Cruz Biotechnology, Inc., Santa Cruz, CA, USA) were used as primary antibodies. Immunoreactive bands were visualized with chemiluminescence using ECL western blot detection reagents (Santa Cruz Biotechnology, Inc., Santa Cruz, CA, USA).

### Animal experiments

The animal experiment was approved by the Institutional Animal Care and Use Committees (IACUC) of Jiangsu Institute of Nuclear Medicine (JSINM2010007). Female nude BALB/c mice (5-6 weeks old) were purchased from Shanghai Laboratory Animals Center (SLAC; Shanghai, China).

MDA-MB-231 tumor model was established by subcutaneously injecting 5×10^6^ cells into the left mammary fat pads of nude mice. When tumors reached the size of 3-5 mm in diameter, mice were randomly divided into three groups (n=6 per group) and were intratumorally injected with PBS (control group), 10^7^ PFU of VG9-IL-24 (VG9-IL-24 group) and 10^7^ PFU of VG9-EGFP (VG9-EGFP group). Tumor growth was followed every other day and the tumor volume was calculated as [(width)^2^ × length] × 0.52^16^. Mice were euthanized when tumors reached their maximal permitted size according to the animal regulations, and Kaplan-Meier survival curves were plotted.

### HE and immunohistochemical staining

Tumors in each group were harvested and fixed in 10 % neutral formalin. After conventional paraffin embedding, hematoxylin and eosin (HE) staining and immunohistochemical staining were performed according to standard protocols. HE staining was used to observe morphological changes in tumor tissue cells, such as karyokinetic, which reflect the proliferation activity of tumor cells.

For immunohistochemical analysis, sections were deparaffinized at 60˚C for 1 h, followed by rehydration, and antigen retrieval using citrate buffer and heating. After treatment with 3 % hydrogen peroxide (H_2_O_2_) to block endogenous peroxidase activity and blocking with PBS containing 1% bovine serum albumin (BSA), sections were washed three times in PBS and incubated for 1 h at room temperature with Ki-67 (1:100, Novus Biologicals, USA ) or monoclonal anti-IL-24 antibody (1:50, Abcam, Cambridge, UK). Slices were then washed in PBS, incubated with the corresponding secondary antibodies for 30 min and detected with diaminobenzidine tetrahydrochloride (DAB) solution. After counterstaining with hematoxylin, slices were dehydrated through a sequence of increasing concentrations of alcohol and cleared in xylene. Images were taken with the microscope (magnification, 400×).

### Statistical analysis

Statistical analysis was performed by SPSS 19.0 software (SPSS Statistics, Inc., Chicago, IL, USA). Data are presented as mean ± standard deviation (SD). One-way ANOVA analyses were employed to compare multiple groups, followed by Tukey's test for two groups. Survival analysis was performed using the method of Kaplan-Meier, and differences between curves were assessed using the log-rank test. The *P* value of less than 0.05 was considered to be statistically significant.

## Results

### Construction and characterization of VG9-IL-24

**Figure 1:**
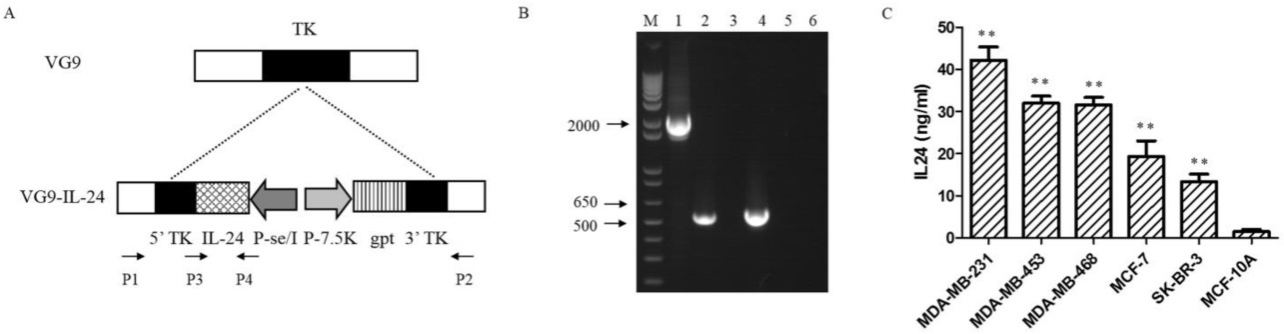
Characterization of VG9-IL-24. (A) Schematic illustration of VG9-IL-24 construction. The IL-24 gene was inserted into TK locus of VG9 strain via homologous recombination. (B) Identification of VG9-IL-24 by PCR analysis. M, 1Kb Plus DNA Ladder (Invitrogen); Lane 1 (VG9-IL-24), P1/P2 primers amplify a 2020-bp fragment across the region of recombination, which confirm absence of TK. The TK positive fragment (534-bp) amplified of VG9 is shown in Lane 3 and negative control (H_2_O) is shown in Lane 5. Lane 2 (VG9-IL-24), P3/P4 primers amplify IL-24 to product a 546-bp band, which is absent in VG9 (Lane 4). Negative control (H_2_O) is shown in Lane 6. (C) Protein concentrations of IL-24 in breast malignant and normal cells. The concentrations of IL-24 protein from all breast cancer cells treated with VG9-IL-24 was higher than that in normal cells (all *P*<0.01). Each bar represents the mean ± SD (n=3). **P <0.01.

VG9-IL-24 was generated by inserting the IL-24 gene into TK locus of vaccinia strain VG9 by homologous recombination (Figure 1A). PCR analysis using specific primers designed were performed to detect the wild-type virus and confirmed the insertion of IL-24 gene (Figure 1B). To further confirm the exogenous IL-24 expression, protein in supernatants and lysates from breast cancer cells and normal cells infected with VG9-IL-24 were harvested and quantified by ELISA. As expected, the concentrations of IL-24 protein from all breast cancer cells treated with VG9-IL-24 was remarkably increased compared with that from normal cells (all *P*<0.01; Figure 1C). Endogenous IL-24 expression was not detected in cells treated with VG9-EGFP or PBS control groups (data not shown).

### Oncolytic activity of VG9-IL-24 *in vitro*

**Figure 2:**
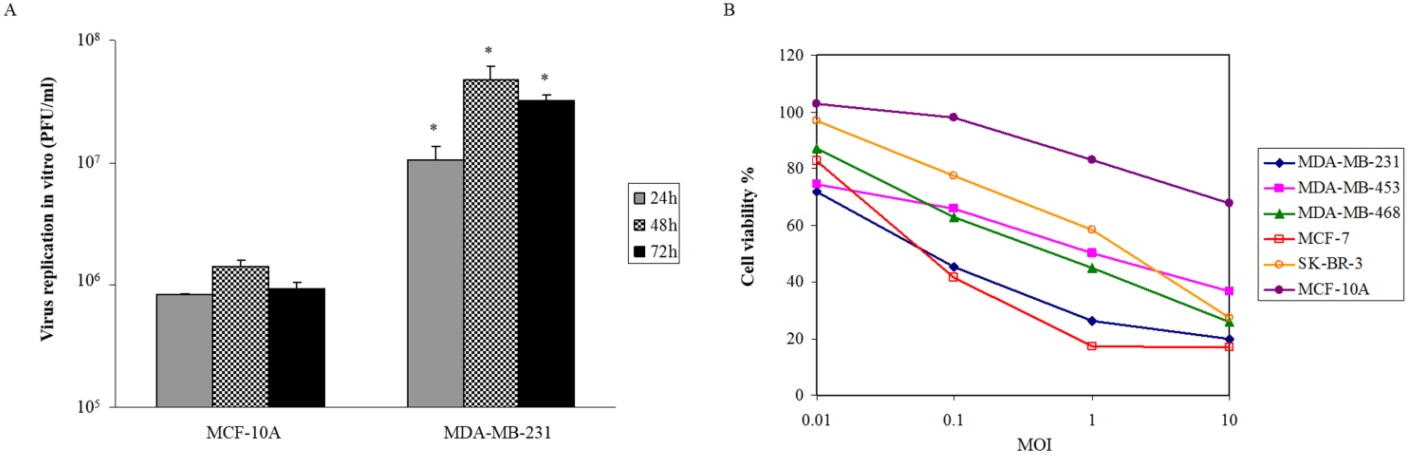
Oncolytic activity of VG9-IL-24. (A) Selective replication of VG9-IL-24 in breast cancer cells. MDA-MB-231 cells and MCF-10A cells in 12-well plates were infected with VG9-IL-24 at 0.1 MOI and samples were collected at indicated times. Virus titers in MDA-MB-231 cells were higher than those in MCF-10A cells at indicated times (all *P*<0.05). Each bar represents the mean ± SD (n=3). *P<0.05. (B) Breast cancer cell lines and normal cells were infected with VG9-IL-24 at various MOIs. After infection for 72 h, cell viability was measured by MTT assay. The cytotoxic effect was obvious on breast cancer cell lines, while little cytotoxicity on normal cells.

Viral proliferation was assessed on breast malignant and normal cells. After infection of VG9-IL-24 at MOI of 0.1, samples were collected at various times. We found that VG9-IL-24 replicated rapidly in MDA-MB-231 cells and the titer was markedly higher than that in MCF-10A cells at indicated times (all *P*<0.05; Figure 2A). To further investigate the selective killing of VG9-IL-24 on tumor cells, various breast cancer cell lines and normal cells were infected with increasing doses of virus and cell viability was assessed by MTT. As shown in Figure 2B, the cytotoxic effect was obvious on breast cancer cell lines with diverse sensitivity to different cell lines, but there was no significant cytotoxicity on normal cells. At MOI of 1, more than 50% of all breast cancer cells were killed. At MOI of 10, less than 20% of MCF-7 and MDA-MB-231 cells survived and about 20%-40% in other breast cancer cell lines. However, VG9-IL-24 had little cytotoxic effect on normal cells.

These results all indicate that VG9-IL-24 can selectively replicate in breast cancer cell lines and has oncolytic potency on tumor cells without significant cytotoxicity to normal cells.

### VG9-IL-24-mediated apoptosis in breast cancer cells

**Figure 3:**
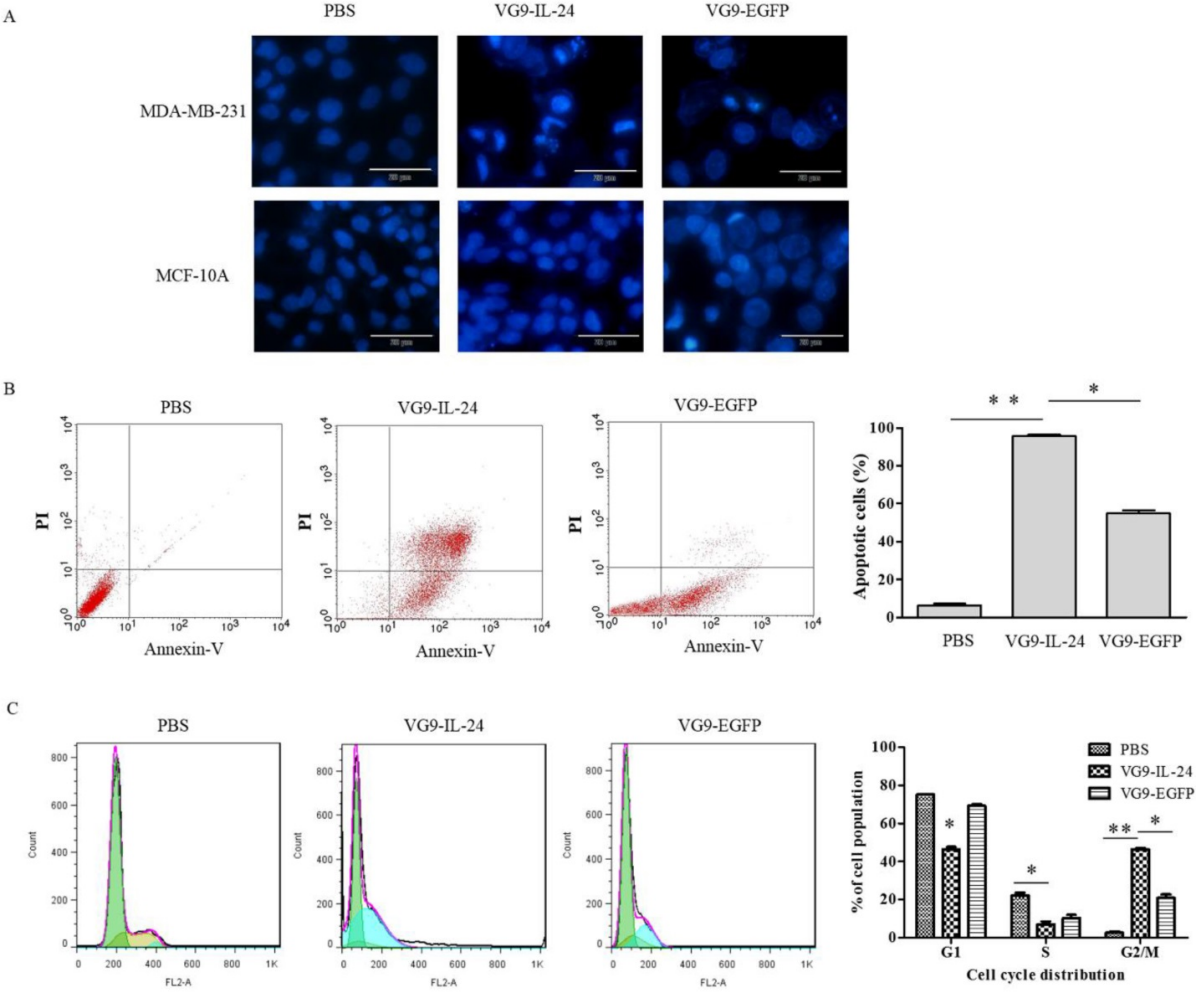
VG9-IL-24 induced apoptosis of breast cancer cells. (A) Cell apoptotic staining by Hoechst 33258. MDA-MB-231 cells and MCF-10A cells treated with PBS, VG9-IL-24 or VG9-EGFP were incubated with Hoechst 33258 for 30 min, and nuclear fragmentation and chromatin clumping were observed in virus-treated groups but not in normal cells. Bar: 20 μm. (B) The percentage of apoptotic cells was determined by flow cytometry. MDA-MB-231 cells treated with PBS, VG9-IL-24 or VG9-EGFP were harvested after 48 h and stained with FITC-labeled Annexin V and PI and immediately analyzed by flow cytometry. The apoptosis ratio of VG9-IL-24 group was significantly higher than that of VG9-EGFP or PBS group (*P*<0.01 compared with PBS group; *P*<0.05 compared with VG9-EGFP group). (C) Cell-cycle analysis by flow cytometry. MDA-MB-231 cells treated with PBS, VG9-IL-24 or VG9-EGFP were harvested after 48 h and stained with PI. Cell cycle distribution was analyzed by flow cytometry and the percentage of cell-cycle phases was analyzed. Higher G2/M proportion of the cell cycle was observed in VG9-IL-24 group compared with PBS or VG9-EGFP group (*P*<0.01 compared with PBS group; *P*<0.05 compared with VG9-EGFP group). Each bar represents the mean ± SD of three independent experiments. *P<0.05; **P <0.01.

VG9-IL-24 induced apoptosis in breast cancer cells was first assessed by Hoechst staining to observe apoptotic morphological changes. As shown in Figure 3A, nuclear fragmentation and chromatin clumping were evidently observed in MDA-MB-231 cells infected with VG9-IL-24 for 48 h, but no obvious apoptotic changes showed in normal cells. No significant changes were observed in any of the cells treated with PBS.

Annexin-V and PI staining assays with flow cytometry were then performed to quantify the apoptosis induction in tumor cells (Figure 3B). After infection with VG9-IL-24, VG9-EGFP and PBS, the apoptosis ratios of MDA-MB-231 cells were (95.62±0.86) %, (54.89±1.52) % and (6.29±0.88) %, respectively. Compared with VG9-EGFP and PBS group, the number of apoptotic cells was obviously increased in VG9-IL-24 group (*P*<0.01 compared with PBS group; *P* <0.05 compared with VG9-EGFP group).

Previous studies have demonstrated that IL-24 is able to induce G2/M cell-cycle arrest in various cancer cell lines 12, 17-19. To determine whether VG9-IL-24 induces G2/M accumulation in MDA-MB-231 cells, cell-cycle phases were analyzed by flow cytometry. Results showed that VG9-IL-24 induced higher G2/M proportion of the cell cycle in MDA-MB-231 cells compared with PBS (*P*<0.01) and VG9-EGFP (*P* <0.05; Figure 3C).

Together, these results indicated that VG9-IL-24 notably inhibited growth, stimulated the apoptosis of breast cancer cells, and arrested breast cancer cells in the G2/M phase.

### VG9-IL-24 regulates PI3K/β-catenin signaling pathway in breast cancer cells

**Figure 4:**
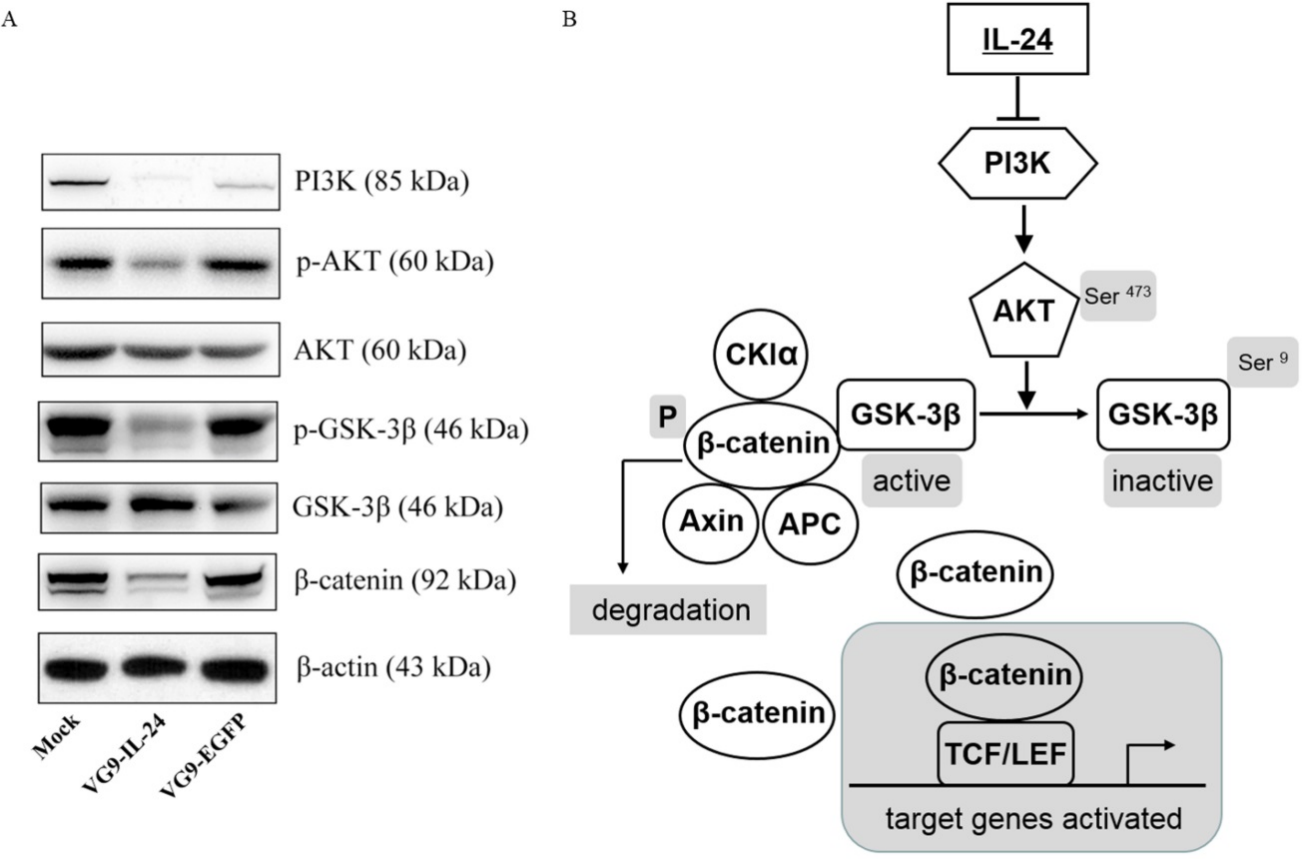
VG9-IL-24 induced apoptosis in breast cancer cells via PI3K/β-catenin signaling pathway. (A) MDA-MB-231 cells treated with PBS, VG9-IL-24, VG9-EGFP for 48 h were harvested, lysed and prepared to be available for western blot analysis. Reduced expressions of PI3K, phosphorylation of Akt and GSK-3β, and β-catenin were observed in VG9-IL-24-treated cells. β-actin was used as a loading control. (B) Schematic illustration of VG9-IL-24 regulation of PI3K/β-catenin signaling pathway.

IL-24 negatively regulating the PI3K/β-catenin pathway was observed in breast cancer cells previously 20. To determine whether the apoptotic effect of VG9-IL-24 on breast cancer cells was related to PI3K/β-catenin signaling pathway, we investigated the activity of VG9-IL-24 on the representative signal proteins of this pathway. As shown in Figure 4A, MDA-MB-231 cells infected with VG9-IL-24 showed reduced levels of PI3K, which resulted in down-regulation of phosphorylation of protein kinase B (Akt, Ser^473^) and subsequent decrease of phosphorylation of glycogen synthase kinase 3β (GSK-3β, Ser^9^) and increase of GSK-3β. As the negative regulator of β-catenin, activated GSK-3β led to decreased level of β-catenin protein due to ubiquitin-proteasome degradation by phosphorylation. Western blotting results suggested that VG9-IL-24 induced apoptosis in breast cancer cells might via PI3K→AKT→GSK-3β→β-catenin pathway (Figure 4B).

### Antitumor effect of VG9-IL-24 *in vivo*

**Figure 5:**
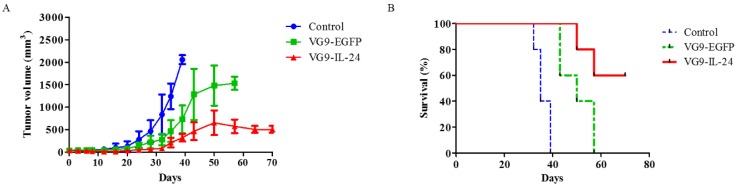
Antitumor effect of VG9-IL-24 in MDA-MB-231 xenograft mouse model. (A) Mean tumor volume in mice treated with PBS (Control), VG9-IL-24, or VG9-EGFP. Tumors developed rapidly in Control and VG9-EGFP groups, while tumor growth was significantly slow in VG9-IL-24 group. n=6 in each group. (B) Kaplan-Meier survival curves for tumor-bearing mice treated with PBS, VG9-IL-24, or VG9-EGFP. Higher survival rate was observed in VG9-IL-24 group compared with Control or VG9-EGFP group. n=6 in each group.

To further evaluate the antitumor effect of VG9-IL-24 *in vivo*, MDA-MB-231 xenograft mouse model was established. When tumors reached 3 to 5 mm in diameter, VG9-IL-24, VG9-EGFP or PBS (control) was intratumorally injected. Results showed that tumors grew progressively in control group, while virus-treated mice exhibited significant suppression of tumor development. By 40 days post-treatment, an increased tumor growth was observed in the VG9-EGFP group, but tumor growth was delayed in the VG9-IL-24 group (Figure 5A). All control mice died within 40 days, however, VG9-IL-24-treated mice survived extended up to 70 days with survival rate of 60% (Figure 5B).

**Figure 6:**
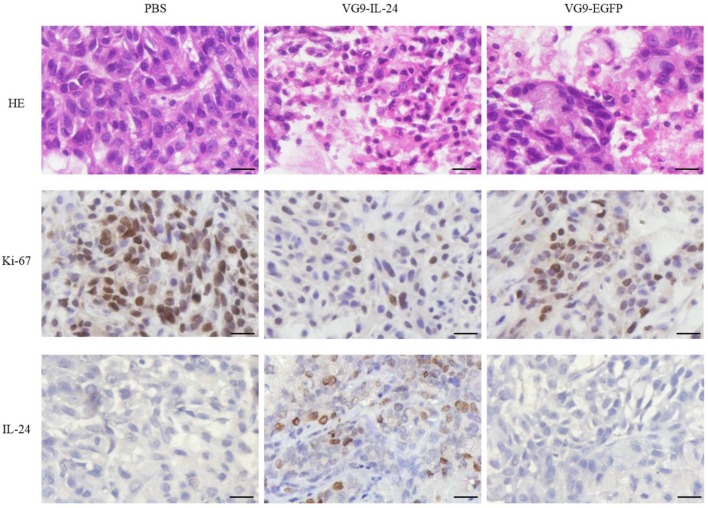
HE and immunohistochemical staining of the tumor tissue. Tumors from the mice treated with PBS (Control), VG9-IL-24, or VG9-EGFP were harvested, formalin fixed and paraffin embedded. Sections were subjected to HE staining and immunohistochemistry for Ki-67 and IL-24. Pyknosis events were obviously appeared in VG9-IL-24 group and decreased positive Ki-67 were also observed in VG9-IL-24 group. Expression of IL-24 was observed in tumors treated with VG9-IL-24. Original magnification, ×400. Bar: 20 μm.

HE and immunohistochemical staining provided further evidence of the antitumor activity of VG9-IL-24. As shown in Figure 6, apparent karyopyknosis, cytoplasm concentration and dissolution of the nucleus were observed in VG9-IL-24 group by HE staining. Immunohistochemical analysis for Ki-67, a tumor cell proliferation marker that positively correlates with prognosis in various malignant tumors, showed that VG9-IL-24 significantly inhibited the proliferation of tumor cells. Immunohistochemical staining also confirmed that IL-24 was stably expressed in tumor tissue from VG9-IL-24-treated group. These results indicated that VG9-IL-24 was able to efficiently generate IL-24 protein, resulting in inhibition of tumor cells proliferation.

## Discussion

Targeted oncolytic vaccinia virus has emerged as a novel therapeutic strategy for cancer in recent years. Due to direct tumor cells lysis by the virtue of selective replication in tumor cells, oncolytic vaccinia virus are potential to treat a wide variety of cancers, including breast cancer. In present study, we constructed the oncolytic vaccinia virus harbored with IL-24 (VG9-IL-24). High and stable expression of IL-24 was available with the replication of vaccinia virus confirmed by ELISA and immunohistochemical staining. The antitumor effects of this recombinant vaccinia virus were significant both *in vitro* and* in vivo*.

Vaccinia virus has been used as a live vaccine in the smallpox eradication with a long history, which making it safe and easy to use. Recently, it as has been used as vaccine against cancer due to multiple favorable attributes. Vaccinia virus replicates and lyses cells rapidly compared with other virus species. It entries into target cells through several membrane fusion pathways within 2 h 21, and spends its entire lifecycle in the cytoplasm, allowing for rapid, efficient replication without negative effects from host cell defenses. The first viral particles produced are secreted from cells within 8 h and infected cells are destroyed 48 h to 72 h after infection 22. With infected cells dying, the viral production decreased. Similarly in our study, VG9-IL-24 replicated rapidly, reaching a maximum within 48 h and the value slightly changed by 72 h.

Many studies and clinic trails have been examined the applicability of several vaccine strains such as Wyeth 23, 24, Copenhagen 25 and Lister 26. However, the researches on Chinese vaccine strain as oncolytic agent were few. The vaccinia virus strain VTT was the most widely used vaccine in China and the biological characteristics have already been studied systematically 27. VTT is less virulent than vaccinia virus strain WR 27, which has been widely used in laboratories and extensively tested in clinical trials. VG9 was derived from VTT by using consecutive plaque-cloning selection and has been demonstrated smaller necrosis area and pock diameter production, less red swelling and lower incidences of fever and hyperpyrexia 13. Although VG9 still had neurotoxicity to a certain extent, the virulence was found to be lower than VTT in various animal models. Therefore, VG9 is supposed to be a promising vaccinia virus vector.

Replication oncolytic vaccinia virus exerts attractive antitumor effects not only through the infection and lysis of cancer cells, but also through expression of various therapeutic transgenes. JX-594, a Wyeth strain armed with GM-CSF, has demonstrated eradication of lung metastases from liver tumors in rabbits 5 and potential antitumor effects both at the injection site and in distant non-injected tumors in advanced hepatocellular carcinoma patients 28, 29. WR strain carrying IL-12 also showed efficient infection in a variety of tumor cell lines and significant reduction in tumor growth 6. The therapeutic gene engineered in this study was IL-24, a novel cancer growth-suppressing and apoptosis-inducing gene, which has been demonstrated efficient and safe in preclinical studies based on adenovirus 30-33. Studies have shown that IL-24 can suppresses cell growth and induce apoptosis in a variety of tumor types 17, 30, 34-37. Our data showed that vaccinia virus-mediated IL-24 exerts strong killing effects on various breast cancer cell lines; of note, it also has the cytotoxic effect on TNBC cells, such as MDA-MB-231, MDA-MB-453 and MDA-MB-468 cells. In contrast, overexpression of IL-24 in normal cells has no significant cytotoxicity 12; similar results were observed in our study that normal breast epithelial cell line MCF-10A exerts no injurious effects.

According to that IL-24 is a prospective tumor suppressor cytokine for multiple cancers, it is worth to explore the mechanism by which IL-24 induces apoptosis selectively in cancer cells. However, the mechanism is complicated and signaling pathways involved are different and seem to be dependent on tumor types 38. Previous studies have revealed that multiple signaling pathways and intracellular molecules play a role in IL-24-mediated apoptosis including activation of the p38 MAPK pathway 39, inhibition of the Wnt/PI3K pathways 20, activation of the Fas-FasL signaling pathway 40, activation of caspase cascade 41, activation of RNA-dependent protein kinase R (PKR) 42 and c-Jun-NH2-kinase 43, downregulation of anti-apoptotic proteins 44, as well as upregulation of pro-apoptotic proteins 30. In this study, we investigated that the apoptotic effect of VG9-IL-24 on breast cancer cells was related to PI3K/β-catenin signaling pathways. Results showed that VG9-IL-24 inhibited expression of oncogenic protein PI3K, thus decreased the phosphorylation of Akt (Ser^473^), which is known as a major downstream mediator of the PI3K pathway. As the downstream target of Akt, phosphorylation of GSK-3β (Ser^9^) downregulated due to Akt kinase activity inhibition, thereby maintaining GSK-3β in its active form which activated the destruction complex (constituted with β-catenin, adenomatous polyposis coli, casein kinase 1α/axin, and GSK-3β) to phosphorylate β-catenin (Ser^33^/Ser^37^/Thr^41^), thus marking it for proteasome-mediated degradation. As a consequence, cytosolic pools of β-catenin reduced and no more translocated into the nucleus to bind the transcription factor T-cell factor/lymphoid enhancer factor (TCF/LEF), leading to inhibiting expression of TCF/LEF-responsive genes which function in cell cycle progression and loss of cell differentiation.

The apoptotic effects of VG9-IL-24 were also investigated by Hoechst staining, flow cytometric analysis, and cell cycle analysis. After VG9-IL-24 treatment, nuclear fragmentation and chromatin clumping in breast cancer cells were observed, apoptotic cell ratio was increased, and accumulation of cells in the G2/M phase of the cell cycle was determined. To further evaluate the antitumor effect of VG9-IL-24 *in vivo*, we established an orthotopic breast cancer model by injecting MDA-MB-231 cells into the mammary fat pads of nude mice. Results showed that tumor growth in VG9-IL-24-treated mice was notably slower compared with VG9-EGFP or control group and the survival rate of VG9-IL-24 group was 60%, which was similar with the other recombinant vaccinia virus carrying IL-12 in previous study 6. HE and immunohistochemical analysis results indicated that tumor cells treated with VG9-IL-24 underwent apoptosis, while apoptosis was not detected in control group or found in only a small amount of tumor cells treated with VG9-EGFP, which was consistent with the results *in vitro* study.

As a novel tumor suppressor cytokine, IL-24 has multifaceted antitumor effects. In addition to selectively suppressing growth and inducing apoptosis of tumor cells, other antitumor features including inhibition of tumor cell invasion and metastasis, anti-angiogenic activity, immune modulatory activity, and “bystander” antitumor activity were also observed 45-48. In present study, we only investigated selective apoptosis induction of VG9-IL-24 on breast cancer cells *in vitro* and xenograft nude mice *in vivo*. To further confirm whether VG9-IL-24 could induce antitumor immunity and “bystander” antitumor effect, immune competent mice models should be established in future study.

Collectively, we constructed a recombinant vaccinia virus VG9-IL-24, which was armed with a potent tumor-suppressing gene IL-24, and demonstrated it exerted significant antitumor effects on breast cancer both *in vitro* and *in vivo*. Beyond direct cell lysis, VG9-IL-24 also induced apoptosis in breast cancer cells without harming normal cells. Our findings suggest that VG9-IL-24 holds a significant promise as an innovative therapy for breast cancer in clinic trails.

## Abbreviations

IL-24: Interleukin-24
VG9: Vaccinia Strain Guang9
VTT: Vaccine Strain Tian Tan
TK: Thymidine Kinase
XGPRT: xanthine-guanine phosphoribosyltransferase
PFU: Plaque-forming Unit
EGFP: enhanced green fluorescent protein
MOI: Multiplicity of Infection
ELISA: Enzyme-linked Immunosorbent Assay
PI: Propidium Iodide
PI3K: Phosphoinositide 3-kinase
AKT: protein kinase B
GSK-3β: glycogen synthase kinase 3β
HE: hematoxylin and eosin
